# Pathological complete response following systemic therapy with carboplatin–paclitaxel plus pembrolizumab for initially unresectable mismatch repair-deficient advanced endometrial cancer: two case reports

**DOI:** 10.1016/j.gore.2026.102093

**Published:** 2026-04-26

**Authors:** Nanami Uetake, Shiho Miura, Yurina Suzuki, Naoki Kojima, Chihiro Kondoh, Koji Horie

**Affiliations:** a Department of Gynecology, Saitama Cancer Center, Saitama, Japan; b Department of Pathology, Saitama Cancer Center, Saitama, Japan; c Department of Medical Oncology, Saitama Cancer Center, Saitama, Japan

**Keywords:** Endometrial cancer, Mismatch repair deficiency (dMMR), Systemic therapy, Chemo-immunotherapy, Pathological complete response (pCR)

## Abstract

**Background:**

Mismatch repair-deficient (dMMR) endometrial cancer is highly sensitive to immune checkpoint inhibition. Although pembrolizumab combined with carboplatin and paclitaxel is now a first-line standard treatment for advanced or recurrent dMMR endometrial cancer, there is limited evidence regarding the effectiveness of this treatment for initially unresectable cancer.

**Case presentation:**

We report two patients with advanced dMMR endometrial cancer who achieved a pathological complete response (pCR) after initial systemic carboplatin–paclitaxel plus pembrolizumab therapy.

Case 1 was a 77-year-old woman diagnosed with FIGO 2008 stage IIIB (cT3bN0M0) grade 3 endometrioid carcinoma who had suspected sigmoid colon and ureter involvement and poorly controlled diabetes mellitus. Primary surgery with complete resection was considered unfeasible because of the anticipated surgical invasiveness and high risk of postoperative complications; therefore, the initial treatment was systemic therapy with carboplatin–paclitaxel plus pembrolizumab. Marked radiologic regression was achieved, bowel resection was avoided at surgery, and a pCR was confirmed.

Case 2 was a 60-year-old woman with FIGO 2008 stage IVB (cT1bN2M1) grade 1 endometrioid carcinoma with suspected metastases in the pelvic, *para*-aortic, and left supraclavicular lymph nodes. After six cycles of systemic carboplatin–paclitaxel plus pembrolizumab therapy, cytoreductive surgery showed no residual viable carcinoma, confirming a pCR.

**Discussion:**

These cases demonstrate that chemo-immunotherapy can induce a pCR in advanced dMMR endometrial cancer and may deepen the treatment response.

**Conclusion:**

Initial carboplatin–paclitaxel plus pembrolizumab therapy may enable less invasive surgery. These findings support the investigation of less invasive approaches for dMMR endometrial cancer.

## Highlights

• Two advanced dMMR endometrial cancers achieved pathological complete response after TC plus pembrolizumab.
• Profound regression enabled avoidance of bowel resection in a case with suspected sigmoid colon invasion.
• Radiographic CR rates in KEYNOTE-868 support deep responses with first-line chemo-immunotherapy in dMMR disease.
• These cases motivate the investigation of less invasive approaches for dMMR endometrial cancer.

## 1 Introduction

The incidence of endometrial cancer is increasing worldwide, and advanced disease remains associated with poor outcomes despite multimodal treatment (Sung, 2021). Molecular classification shows that mismatch repair-deficient (dMMR)/microsatellite instability-high (MSI-H) disease is relatively common in endometrial cancer and is present in approximately 30% of cases (Addante, 2024). Such dMMR tumors comprise an immunogenic subgroup with marked sensitivity to immune checkpoint inhibition.

Evidence for the effectiveness of immune checkpoint inhibition in the treatment of recurrent disease comes from two distinct trial populations. The KEYNOTE-158 trial was a tumor-agnostic study that enrolled patients with previously treated MSI-H/dMMR solid tumors, in which pembrolizumab monotherapy showed durable activity and an objective response rate of 48% in the endometrial cancer cohort (O’Malley, 2022). In contrast, the KEYNOTE-775 trial enrolled only patients with recurrent endometrial cancer after prior platinum-based therapy and demonstrated improved progression-free survival (PFS) with pembrolizumab plus lenvatinib versus chemotherapy (median PFS 6.6 vs. 3.8 months) (Makker, 2022).

More recently, the KEYNOTE-868 (NRG-GY018) trial reported a significant improvement in PFS when pembrolizumab was added to carboplatin–paclitaxel as a first-line therapy for advanced or recurrent endometrial cancer (Eskander, 2023). When upfront cytoreductive surgery is unfeasible, initial systemic therapy may be considered; however, carboplatin–paclitaxel alone yields low clinical complete response (cCR) rates of 0%–13.7% (Khouri, 2019, Philp, 2021, de Lange, 2019, Eskander, 2025). Herein, we report two cases in which patients with advanced dMMR endometrial cancer achieved a pCR with carboplatin–paclitaxel plus pembrolizumab therapy.

## 2 Case presentations

### 2.1 Case 1

**Fig. 1:**
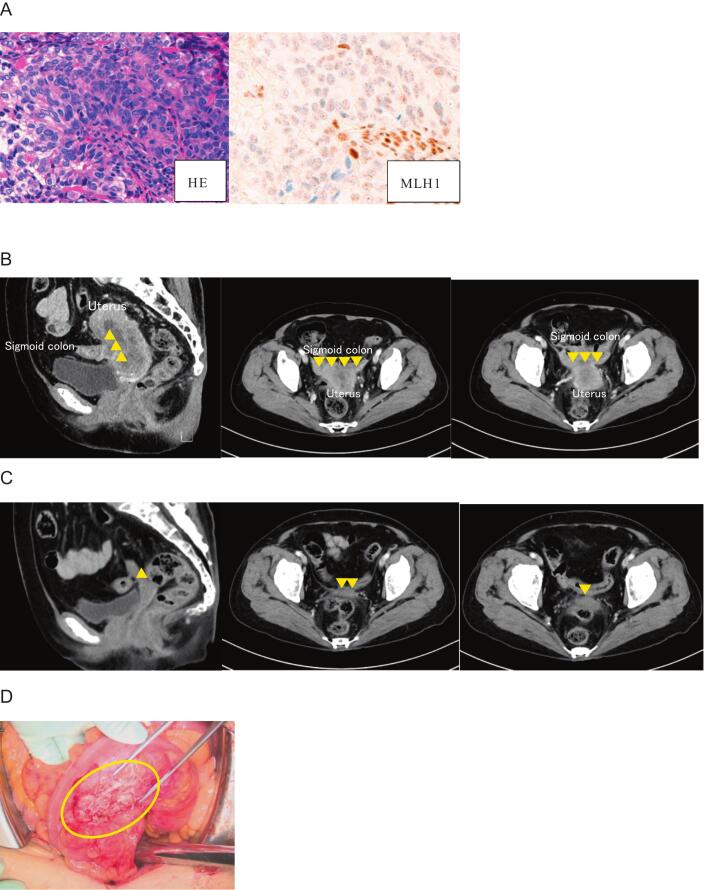
Imaging findings in case 1. (A) The tumor was diagnosed as a grade 3 endometrioid carcinoma based on an endometrial biopsy (×25 magnification; hematoxylin and eosin stain). Immunohistochemistry showed a complete loss of MLH1 expression (×40 magnification). (B) Baseline computed tomography (CT) shows that the uterus is almost entirely replaced by tumor, with suspected invasion (yellow triangle) into the sigmoid colon. (C) CT after three cycles of carboplatin–paclitaxel plus pembrolizumab shows marked shrinkage of the uterine tumor and reduction of the suspected sigmoid colon invasion (yellow triangle). (D) Intraoperative frozen section analysis reveals that tissue sampled from the surface of the sigmoid colon after adhesiolysis (yellow circle) shows only smooth muscle and hyalinized tissue. Therefore, bowel resection was avoided. (For interpretation of the references to colour in this figure legend, the reader is referred to the web version of this article.)

A 77-year-old woman with diabetes mellitus presented with postmenopausal bleeding. Endometrial biopsy revealed grade 3 endometrioid carcinoma, clinically staged as FIGO 2008 stage IIIB (cT3bN0M0). Immunohistochemistry confirmed dMMR via MutL homolog 1 (MLH1) loss (via hypermethylation) (Fig. 1A). The baseline serum cancer antigen 125 (CA125) concentration was 56.3 U/mL. Computed tomography (CT) suggested suspected invasion of the sigmoid colon (Fig. 1B) and ureteral involvement in the parametrium. Surgery was initially not performed because the disease was deemed unresectable. Diagnostic laparoscopy and confirmatory biopsy were not performed prior to treatment initiation because the disease was assessed based on radiologic findings and systemic therapy was prioritized. To allow the safe continuation of systemic therapy (including corticosteroid-containing antiemetic regimens), the glycemic control was optimized and systemic therapy comprising carboplatin (area under the blood concentration time curve 5), paclitaxel (175 mg/m^2^), and pembrolizumab (200 mg) was administered every 3 weeks.

After three treatment cycles, CT demonstrated tumor regression (Fig. 1C) with a decrease in the CA125 concentration to 19.5 U/mL. However, the HbA1c increased from 8.1% to 8.5%, and three additional treatment cycles were administered while the glycemic control was improved with insulin. During treatment cycles four to six, the paclitaxel dose was reduced to 135 mg/m^2^ owing to grade 2 peripheral neuropathy. After six treatment cycles, the CT findings were similar to the findings after three cycles and the HbA1c had improved to 6.9%.

As the unexpectedly deep radiologic response suggested that complete resection was feasible, open surgery comprising abdominal total hysterectomy, bilateral salpingo-oophorectomy, partial omentectomy, and pelvic lymph node biopsy was performed to mitigate potential tumor-related complications at 4 weeks after the last pembrolizumab dose. The pelvic lymph node biopsy and partial omentectomy were performed because enlarged lymph nodes and a suspected omental lesion were identified intraoperatively, suggesting metastatic involvement. Although the uterus was densely adherent to the sigmoid colon, careful dissection enabled separation without bowel resection (Fig. 1D). Frozen section analysis of tissue from the sigmoid colon surface showed muscular and hyalinized tissue without residual tumor. Therefore, sigmoid colectomy was not performed. The final pathological examination confirmed a pCR. As no residual disease was identified, the patient is being followed up without maintenance pembrolizumab. The patient shows no evidence of disease at 9 months postoperatively.

### 2.2 Case 2

**Fig. 2:**
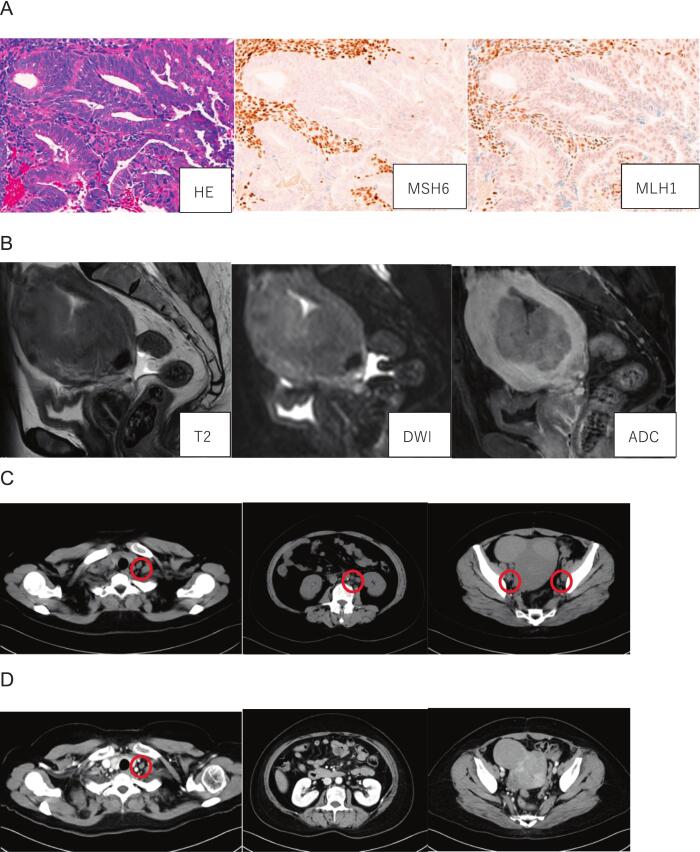
Imaging findings in case 2. (A) Grade 1 endometrioid carcinoma was detected by endometrial biopsy (×20 magnification; hematoxylin and eosin stain). On immunohistochemical analysis, the tumor cells were negative for MSH6 and MLH1/PMS2 staining (PMS2 not shown) (×20 magnification). (B) Baseline magnetic resonance imaging shows myometrial invasion in approximately half of the uterus. (C) Baseline CT shows enlarged left supraclavicular, *para*-aortic, and pelvic lymph nodes (red circles). (D) CT after three cycles of carboplatin–paclitaxel plus pembrolizumab revealed shrinkage of the left supraclavicular lymph node (red circle) and complete resolution of the other lesions. (For interpretation of the references to colour in this figure legend, the reader is referred to the web version of this article.)

A 60-year-old woman presented with postmenopausal bleeding and an endometrial biopsy confirmed grade 1 endometrioid carcinoma. Initial CT and positron emission tomography (PET)-CT revealed suspected metastases in the pelvic, *para*-aortic, and left supraclavicular lymph nodes, consistent with FIGO 2008 stage IVB (cT1bN2M1) (Fig. 2B-C). The initial maximum standard uptake values of these three regions were 17.90, 26.14 and 8.64, respectively. Biopsy of the supraclavicular lymph node was not performed because metastatic involvement was clinically inferred based on the imaging findings. Immunohistochemistry revealed loss of MLH1, postmeiotic segregation increased 2 (PMS2), and MutS homolog 6 (MSH6) expression, confirming dMMR (Fig. 2A). The baseline CA125 concentration was 129.7 U/mL. Systemic therapy comprising carboplatin (area under the blood concentration time curve 5), paclitaxel (175 mg/m^2^), and pembrolizumab (200 mg) was administered every 3 weeks.

After three treatment cycles, CT showed marked regression of most metastatic lesions, although the left supraclavicular lymph node remained enlarged (Fig. 2D). PET-CT was not performed, as contrast-enhanced CT demonstrated a clear reduction in tumor burden and was considered sufficient for clinical assessment. The CA125 concentration decreased to 19.9 U/mL, and a repeat endometrial biopsy to evaluate the treatment response showed no residual carcinoma. After three additional treatment cycles, the CA125 concentration further decreased to 17.7 U/mL and the left supraclavicular lymph node had shrunk.

At 4 weeks after the last pembrolizumab dose, open surgery comprising abdominal total hysterectomy, bilateral salpingo-oophorectomy, and pelvic lymph node biopsy to assess for residual tumor cells was performed to prevent future tumor-related symptoms. No macroscopic disease was observed intraoperatively, and the final pathological examination confirmed a pCR. Pembrolizumab maintenance therapy was started 6 weeks after surgery based on the clinical indication for stage IVB disease. At 8 months postoperatively, she remains disease-free.

## 3 Discussion

The present report describes two patients with advanced dMMR endometrial cancer who achieved a pCR following systemic therapy with carboplatin–paclitaxel plus pembrolizumab, illustrating the depth of response achievable with chemo-immunotherapy in this molecular subgroup. dMMR tumors are highly immunogenic because of the high tumor mutational burden and abundant neoantigens, conferring sensitivity to immune checkpoint inhibition. The prevalence of MSI-H/dMMR tumors also differs across cancer types. A real-world dataset of unresectable or metastatic solid tumors found that the MSI-H type is more prevalent in endometrial cancer than in colorectal cancer (16.85% vs. 3.78%) (Akagi, 2021), supporting the use of immunotherapy-driven treatment de-escalation for endometrial cancer.

In the KEYNOTE-868 trial, carboplatin–paclitaxel plus pembrolizumab significantly improved PFS versus chemotherapy alone in both the dMMR cohort (hazard ratio 0.30; 12-month PFS 74% vs. 38%) and the mismatch repair-proficient (pMMR) cohort (hazard ratio 0.54; median PFS 13.1 vs. 8.7 months) (Eskander, 2023). Furthermore, among patients with measurable disease, clinical complete responses assessed according to RECIST v1.1 were numerically more frequent with pembrolizumab plus chemotherapy than with chemotherapy alone (dMMR: 31.6% vs. 13.7%; pMMR: 14.3% vs. 8.4%) (Eskander, 2025). Our cases provide pathological confirmation that chemo-immunotherapy can eradicate viable tumors in certain patients with advanced dMMR endometrial cancer. Case 1 showed regression of the suspected sigmoid colon invasion with avoidance of bowel resection, while Case 2 demonstrated a pCR despite distant lymph node metastases. Historically, systemic therapy with carboplatin and paclitaxel alone has resulted in a low incidence of a clinical CR (cCR) in patients with advanced endometrial cancer (0%–13.7%) (Khouri, 2019, Philp, 2021, de Lange, 2019, Eskander, 2025), underscoring the potential additive effect of immune checkpoint blockade (Eskander, 2025).

In both patients, the initial treatment strategy consisted of systemic therapy followed by surgery primarily intended for symptom palliation and local disease control. This approach was chosen because upfront complete resection was considered unfeasible owing to the anticipated surgical invasiveness and high risk of postoperative complications. However, the marked tumor response observed during chemo-immunotherapy resulted in a substantial reduction in tumor burden, thereby expanding the potential extent of surgical resection. Although marked tumor regression was observed radiologically after systemic therapy, the presence of residual viable disease could not be excluded preoperatively. Surgery was therefore performed with the aims of symptom palliation, local disease control, resection of suspected residual disease, and pathological assessment of treatment response, including assessment of radiographically involved lesions. As a result, the surgical approach was determined intraoperatively on a case-by-case basis, based on the real-time assessment of disease extent and resectability. Pathological examination ultimately revealed a pCR in both cases. Although neoadjuvant chemotherapy was not initially planned in these two cases, the treatment sequence was analogous to systemic therapy followed by interval surgery. Patients with advanced endometrial cancer in whom upfront complete resection is considered unfeasible and the primary tumor is left in situ may develop local symptoms or complications such as bleeding, pelvic pain, infection, or compression-related symptoms; however, the incidence of these complications has not been well quantified. Because surgery and radiotherapy for symptom control and local disease control are recognized as clinically relevant treatment options, the management of residual primary tumors remains an important issue in clinical practice.

The present cases suggest that the role of surgical intervention may warrant re-evaluation in selected patients with advanced dMMR endometrial cancer who achieve a deep response to systemic chemo-immunotherapy. In dMMR rectal cancer, Cercek et al. reported a 100% cCR rate after neoadjuvant PD-1 blockade with non-operative management under surveillance (Cercek, 2022). In a broader dMMR solid-tumor cohort, cCR was achieved in all patients with rectal cancer (49/49) and in 65% (35/54) of those with non-rectal tumors, with a 2-year recurrence-free survival rate of 92% while preserving the option for curative resection (Cercek, 2025). However, the applicability of these strategies to endometrial cancer remains uncertain. Therefore, any consideration of modifying surgical management in endometrial cancer should be approached with caution and requires validation in prospective studies. Continued follow-up and accumulation of similar cases are essential to better define the role, timing, and extent of surgery in this setting.

The optimal strategy for post-treatment surveillance and the duration of immunotherapy in patients with advanced dMMR endometrial cancer who achieve a deep or complete response remain uncertain. In the present cases, follow-up was primarily based on imaging studies and tumor marker assessment, and additional modalities such as PET-CT or liquid biopsy approaches were not routinely employed. While emerging techniques, including circulating tumor DNA and circulating tumor cell assays, may offer further insight into minimal residual disease, their clinical utility in endometrial cancer has not yet been established. Therefore, careful longitudinal follow-up and accumulation of further clinical data are required to better define appropriate surveillance strategies and treatment duration in this setting.

This study has several limitations. First, pathological confirmation of malignancy was not performed at all suspected metastatic sites. Neither diagnostic laparoscopy nor confirmatory biopsy was undertaken prior to treatment initiation in case 1, and biopsy of the supraclavicular lymph node was not performed in case 2. Therefore, the initial disease extent was based primarily on radiologic assessment. Second, the follow-up duration remains relatively short, which limits the ability to draw conclusions regarding the durability of response. Accordingly, these findings should be interpreted with caution, and further accumulation of cases with longer follow-up and more comprehensive diagnostic evaluation is warranted.

## 4 Conclusion

This case report demonstrates that systemic therapy with carboplatin–paclitaxel plus pembrolizumab can induce a pCR in patients with advanced dMMR endometrial cancer. In the present two cases, the marked tumor response observed with chemo-immunotherapy was associated with changes in surgical management and may have contributed to a reduction in surgical extent.

However, given the limited number of cases and the short follow-up periods, these findings should be interpreted with caution. Further studies are required to clarify the clinical significance of these observations.

Written informed consent was obtained from the two patients for publication of this case report and accompanying images.

## Declaration of Generative AI and AI-assisted technologies in the writing process

During the preparation of this manuscript, the authors used ChatGPT (GPT-5.2 by OpenAI) and Gemini (Gemini 3 Flash by Google) to improve the readability and linguistic quality of the text. After using these services, the authors reviewed and edited the content as needed and take full responsibility for the accuracy and integrity of the final publication.

## CRediT authorship contribution statement

**Nanami Uetake:** Writing – original draft, Visualization, Investigation, Conceptualization. **Shiho Miura:** Writing – review & editing, Investigation, Conceptualization. **Yurina Suzuki:** Writing – review & editing, Conceptualization. **Naoki Kojima:** Visualization, Resources, Conceptualization. **Chihiro Kondoh:** Writing – review & editing, Conceptualization. **Koji Horie:** Writing – review & editing, Supervision, Conceptualization.

## Informed consent

Written informed consent was obtained from the two patients for the publication of this case report.

## Funding

This research did not receive any specific grant from funding agencies in the public, commercial, or not-for-profit sectors.

## Declaration of competing interest

The authors declare that they have no known competing financial interests or personal relationships that could have appeared to influence the work reported in this paper.
